# The irregular breathing effect on target volume and coverage for lung stereotactic body radiotherapy

**DOI:** 10.1002/acm2.12663

**Published:** 2019-06-17

**Authors:** Chia‐Hsin Pan, An‐Cheng Shiau, Kai‐Chiun Li, Shu‐Hui Hsu, Ji‐An Liang

**Affiliations:** ^1^ Department of Radiation Oncology China Medical University Hospital Taichung Taiwan; ^2^ Department of Biomedical Imaging and Radiological Sciences National Yang‐Ming University Taipei Taiwan; ^3^ Department of Biomedical Imaging and Radiological Science China Medical University Taichung Taiwan; ^4^ Department of Radiation Oncology Montefiore Medical Center Bronx New York; ^5^ Department of Medicine China Medical University Taichung Taiwan

**Keywords:** stereotactic body radiotherapy, IMRT, VMAT, 4D CT, irregular breathing pattern, organ motion effects

## Abstract

The major challenge in treating a mobile target is obtaining the temporal and spatial information imaging and treatment details. This phantom study quantitatively evaluates the geometric and dosimetric effects of various treatment techniques under different respiratory patterns. The regular motion model was a sinusoidal waveform with a longitudinal range of ±1.5 cm and a period of 4 sec, while irregular motion models were generated by extracting signals from clinical cases. Helical CT for a static target and 4D CT with retrospective sorting were acquired. Phase bin, maximum, and average intensity projection (MIP and AIP) CT datasets were reconstructed. RapidArc and IMRT plans were generated on static and moving target CT datasets with different motion patterns using the phase‐based gating and nongating treatment. Dose measurements were performed using EBT3 films. Dose profile and gamma analysis (±3%/1 mm criteria) were used for dose comparisons. For the irregular motions, internal target volume variations between AIP and MIP datasets (AIP/MIP) had slight differences (−6.2% to −7.7%) for gated plans, and larger differences (−12.3% to −15.2%) for nongated plans. Dosimetric measurements showed a high gamma passing rate (>98.5%) for the static plan in the target region, while the AIP and MIP gated plans had average passing rates of 92.2% ± 5.7% and 85.8% ± 9.5%, respectively. Nongated plans had significantly lower and deviated passing rates, while the AIP and MIP plans had passing rates of 43.6% ± 22.2% and 66.7% ± 28.2%, respectively (*p* < 0.05). Lung stereotactic body radiotherapy treatment delivered with the gated technique did not compromise the gross tumor volumes coverage, and was insensitive to the breathing irregularities and plan techniques. Adequate margins should be accounted to cover the mis‐gating effect when using the phase‐based gating under irregular motion.

**Abbreviations**
4D CT: Four‐dimensional computed tomography; A/P; anterior/posterior; AAA; Analytical Anisotropic Algorithm; A‐I; amplitude irregular; AIP; Average Intensity Projection; C/C; cranial/caudal; CTV; clinical target volume; D_p_
; prescribed dose; GI; gradient index; GTV; gross tumor volume; HU; Hounsfield unit; IMRT; Intensity‐Modulated Radiation Therapy; ITV; internal target volume; ITV_th_
; theoretical ITV; MIP; Maximum Intensity Projection; NSCLC; non‐small cell lung cancer; OAR; organ at risk; P + A‐I; period and amplitude irregular; P‐I; period irregular; PIV; prescription isodose volume; PTV; planning target volume; RA; Rapidarc; SABR; stereotactic ablative radiotherapy; SBRT; Stereotactic body radiotherapy; S‐I; slight irregular; TPS; treatment planning system.

## 1 INTRODUCTION

Stereotactic body radiotherapy (SBRT), also known as stereotactic ablative radiotherapy (SABR), is capable of delivering highly conformal radiation doses to diseases such as early‐stage non‐small cell lung cancer (NSCLC) and reported to provide high local control with limited toxicity.^1^, ^2^, ^3^ However, the major challenge in treating a mobile target is obtaining the temporal and spatial information imaging and treatment details. Respiratory motion is patient specific,^4^ and a more crucial issue is irregular breathing causes the mobile target motion pattern to vary. Unfortunately, irregular breathing is a common clinical situation. Although coaching could improve the breathing pattern reproducibility, it could not totally avoid target irregular motion during the imaging and treatment process.

The impact due to motion pattern variations could be dosimetric and geometric. Four‐dimensional computed tomography (4D CT) is widely used to obtain the temporal and spatial information for a moving target. A 4D CT dataset is generally retrospective sorting with phase binning or amplitude binning. Amplitude binning is more accurate, but it is more sensitive to irregular breathing which can cause image gaps. Phase binning displays no gaps but suffers artefacts due to mis‐binning.^5^ To avoid missing slices from amplitude binning under different irregular breathing patterns, phase binning was used in this study. Maximum intensity projection (MIP) and average intensity projection (AIP) images created from the 4D CT phase bin datasets are usually used for treatment planning on a moving target.^6^ However, irregular breathing motion in 4D CT could cause a mis‐binning process and result in geometric variations, therefore significantly affecting target delineation accuracy.^5^, ^7^, ^8^, ^9^, ^10^ The main factors that affect dose delivery accuracy for a moving target are the interplay effect and the variations in patient breathing patterns during treatment. The interplay effect on an intensity‐modulated dose delivery technique has been studied with proper margin and the full target motion range included, this effect is much less for clinical target volume (CTV) compared with planning target volume (PTV).^11^, ^12^, ^13^, ^14^, ^15^ However, baseline shifts and irregular motion patterns have been shown to exert a remarkable influence on dose delivery accuracy.^11^, ^16^ For a realistic tumor motion treatment study, Court et al.^17^ used rapid prototyping techniques to create tumor models with realistic shapes and to drive a phantom motion with actual patient motion trajectory. Their study also found that the dose deviations averaged out after several fractions. However, how the different irregular breathing pattern conditions affect the dose delivery accuracy were not evaluated in most of these studies.

This phantom study quantitatively evaluates the geometric and dosimetric effects of various treatment techniques under different respiratory patterns. These techniques include a comparison between IMRT and rapid arc (RA) plans, gated and nongated dose delivery techniques, regular and irregular breathing patterns, and different CT datasets. Radiochromic EBT3 films were used for dose measurements.

## 2 MATERIALS AND METHODS

### 2.1 Phantom and respiratory motion models

**Figure 1:**
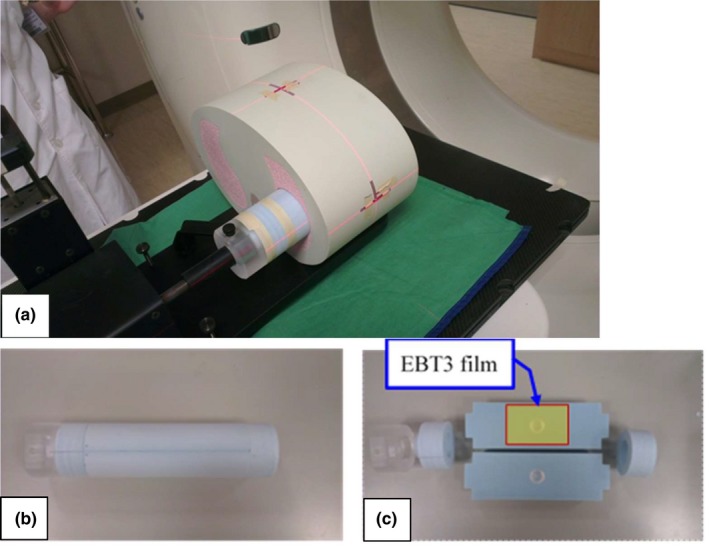
Anthropomorphic thorax phantom (CIRS Dynamic Thorax Phantom, CIRS Inc., Norfolk, VA, USA) (a). The motion rod (b) with two 20 mm diameter and soft‐tissue equivalent hemispherical target embedded in a Styrofoam (c) for use with the phantom.

An anthropomorphic thorax phantom (CIRS Dynamic Thorax Phantom, CIRS Inc., Norfolk, VA, USA) was used [Fig. 1(a)]. Two customized soft‐tissue equivalent hemispherical targets (with 20 mm diameter) were embedded in a Styrofoam motion rod for use with the phantom [Figs. 1(b) and 1(c)]. The Real‐Time Position Management Respiratory Gating System (RPM™) (Varian Medical Systems, Palo Alto, CA, USA) was used to infer target motion.

**Figure 2:**
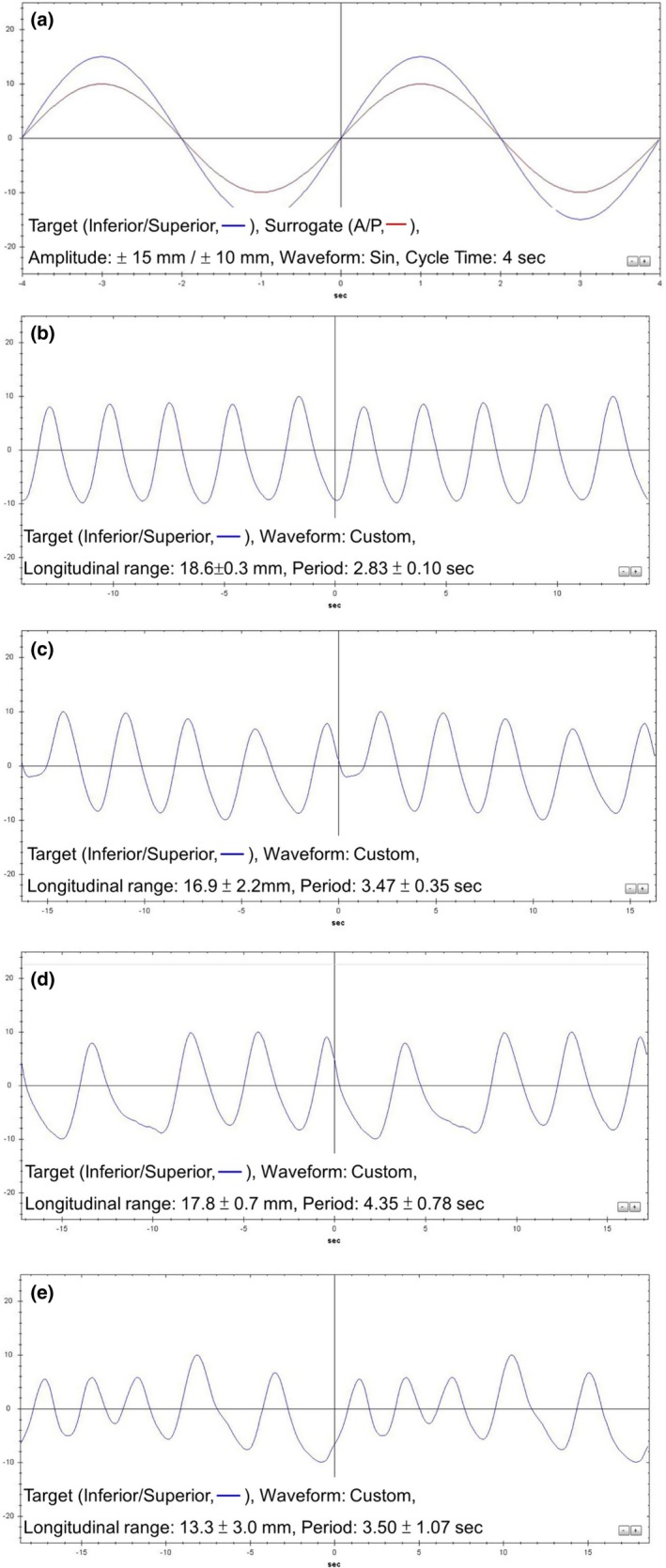
The regular (a) and irregular motion patterns: slightly irregular (b), amplitude irregular (c), period irregular (d) and both of amplitude and period irregular (e) patterns.

Regular and irregular motion models were created and imported into this phantom for imaging, planning, and treatment delivery. The regular motion model was a sinusoidal waveform with a longitudinal range of ±15 mm and a period of 4 sec. The irregular motion models were generated by extracting the RPM signals of different amplitude degrees or period variations from different patients. The irregular motion models included (a) slightly irregular, (b) amplitude irregular, (c) period irregular, and (d) period and amplitude irregular. The longitudinal range and period for irregular motion models were listed in Table 1, and the waveforms were shown in Fig. 2.

**Table 1:** Irregular motion models.

Motion model	Motion pattern	Longitudinal range (mm)	Period (sec)
Slightly irregular (S‐I)	Period and amplitude variation ≤10%	18.6 ± 0.3	2.83 ± 0.10
Amplitude irregular (A‐I)	Amplitude variation ≥30%	16.9 ± 2.2	3.47 ± 0.35
Period irregular (P‐I)	Period variation ≥30%	17.8 ± 0.7	4.35 ± 0.78
Period and amplitude irregular (P+A‐I)	Period and amplitude variation ≥30%	13.3 ± 3.0	3.50 ± 1.07

### 2.2 CT imaging

Siemens CT (SOMATOM Definition AS 64‐slice Configuration, Erlangen, Germany) was used to acquire the helical CT for a static target, with retrospective sorting 4D CT for a motion target in regular and all irregular motion patterns with a slice thickness of 1.0 mm. Phase bin CT datasets were generated in 10 phases and 20 phases, and MIP and AIP CT datasets were reconstructed from the 4D CT datasets to evaluate the target position accuracy and geometric distortion.

### 2.3 Treatment planning and delivery systems

All measurements were conducted on a Varian TrueBeam (Varian Medical Systems, Palo Alto, CA) machine equipped with a Millennium 120 leaf MLC. Beam energies of 6 MV flattening‐filter‐free (FFF) beams were used. Varian Eclipse treatment planning system (TPS) with algorithms of Analytical Anisotropic Algorithm (AAA, v13.6.23) for dose calculation and photon optimizer (PO, v13.6.23) for plan optimization were used. The dose calculation grid size was 2.5 mm for all calculations.

CT images were imported into the TPS for contouring and planning. The gross tumor volumes (GTV), a 20 mm diameter spherical target, were delineated on static and phase bin CTs. For MIP and AIP images, the internal target volumes (ITVs) were defined as the GTV envelope delineated in the selected respiratory cycle phases. The ITV in the nongated plans included all GTV phases. In the gated plans, the ITV included only the selected GTV phases (interval of 30%–70% at the end‐exhalation phases). An automatic contouring function delineated the GTVs and ITVs, which then modified manually according to a clinical procedure. Afterwards, a 5 mm uniform margin was added to the GTV or ITV to generate the PTV.

Treatment plans with the IMRT and RA techniques, and with the gated and nongated dose delivery, methods were generated. All plans were optimized with at least 95% of the PTV encompassed by the prescribed dose (*D_p_*, 6 Gy), and at least 99% of the PTV receiving doses higher than 90% of the *D_p_*. The critical organ dose‐volume limits and dose conformity and gradient quality parameters were controlled according to the RTOG 0915 report.^18^ The “high dose spillage” in this phantom study was much less than the criteria in this report, and had a value <1%. According to the planning optimization clinical procedure, a tighter dose constraint to the organ at risk (OAR) was set during planning if a relatively low dose to the OAR was achievable.

The jaw tracking technique, dynamic sliding window, and a nominal dose rate of 1200 MU/min were used for the plans. The IMRT plans consisted of seven coplanar beams at gantry angles of 310°, 350°, 30°, 70°, 110°, 150°, and 180°. For each RA plan, two coplanar arcs with gantry rotations of 179°−310° (CCW) and 310°−179° (CW) were adopted. The collimator angles of 15° and 105° were used for RA plans, and no collimator rotation was used for IMRT plans.

Including the respiratory patterns, 34 plans were generated and measured. Before each plan irradiation, a half‐fan full rotation CBCT scan was performed for position verification. The AIP CT was used for image‐guided in the motion target plan registration.

### 2.4 Geometrical and dosimetric analysis

Radiochromic EBT3 film with high spatial resolution, near‐tissue equivalence and weak energy dependence were proven a viable tool for external beam dosimetry.^19^, ^20^ All films used in this study were from the same lot number. Each film sheet of 25 × 20 cm^2^ was cut into smaller pieces, size of 4 × 4 cm^2^ for dose‐response calibrations, and 10 × 5 cm^2^ for plan dose measurements [Fig. 1(c)]. The red channel data with 16 bit digital information (pixel value, PV) were extracted and processed using the public domain software ImageJ Version 1.43 (National Institute of Health, Bethesda, MD) for dose profile comparisons, and using the FilmQA Pro software (Ashland Inc)^21^ for plane dose comparisons. The net optical density (netOD) was calculated by subtracting the nonirradiation OD value:

(1) $$\text{netOD}={\text{OD}}_{\exp}-{\text{OD}}_{\text{Bg}}=\log_{10}\left(PV_{\text{Bg}}/PV_{\exp}\right)$$

where *PV*_Bg_ and *PV*_exp_ are the pixel value for the unexposed (background) and exposed film piece, respectively. The sensitometric curve of EBT3 film was fitted with a third order polynomial function (netOD‐to‐dose polynomial function) and applied to each measurement film respectively to convert the dose.

A gamma evaluation^22^ was used with gamma‐index criteria of 3% (dose difference) and 1 mm (distance to agreement). The gamma analysis region was manually adjusted to the target area encompassing the GTV for plane dose comparisons. Passing rates for 4D CT types (MIP and AIP), treatment planning techniques (IMRT and RA), dose delivery methods (gated and nongated), and breathing patterns (regular and irregular) were evaluated. The statistical comparisons between different planning techniques were performed using a paired *t*‐test method. *P* values of ≤0.05 were considered significant.

In the static and regular motion geometrical analysis, the GTV and ITV were compared to the real target ball volume and theoretical ITV (*ITV_th_*), respectively. The *ITV_th_* was calculated as:

(2) $$ITV_{\mathrm{th}}=4/3\pi r^{3}+L\times \pi r^{2}$$

where *r* is the ball radius and *L* is the longitudinal target motion range.^23^ Image artifacts and target center positions in different phases for the 10‐phase and 20‐phase CT images were evaluated under the regular motion condition. The target motion velocity in a sinusoidal waveform was calculated as:

(3) $$V\left(p\right)=-2\pi A/T\times \sin\left(2\pi p/100\right)$$

where *p* is the phase (%), where A is the amplitude, and T is the period.^24^ For a regular motion pattern in this study, the maximum motion velocity was 23.56 mm/sec.

For the different irregular breathing patterns, the ITVs varied depending on the irregular breathing patterns, and calculation could not obtain the *ITV_th_*. Accordingly, the ITVs in the MIP and AIP CT images were compared to each other.

## 3 RESULTS

### 3.1 Geometrical variations

**Figure 3:**
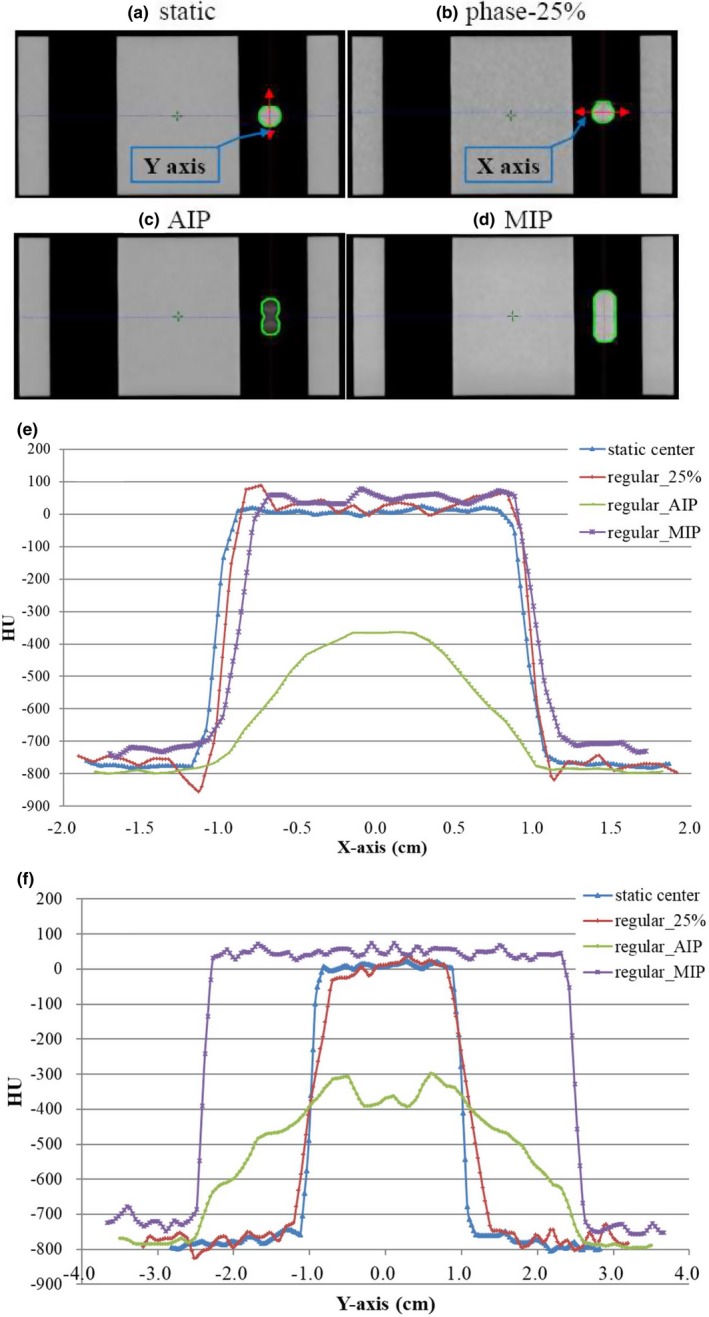
The coronal images of different CT datasets (a–d) and the target Hounsfield Unit (HU) distributions in static and regular motion pattern for phase CT (25%), maximum intensity projection and average intensity projection CT datasets on X‐ (e) and Y‐axis (f).

The target Hounsfield unit (CT number) distributions in static and regular motion patterns for phase CT (25%), MIP and AIP CT datasets on X‐ and Y‐axis are shown in Fig. 3. Compared with static CT, MIP images showed slightly higher, ~50 HUs in the target region. By contrast to MIP images with a homogeneous HU distribution in the target region, AIP images had a much lower HU value and the HU was distributed as the time position function of the motion pattern (−780 HUs to −350 HUs).

The target center position variations for a regular motion pattern in 10‐phase and 20‐phase CT images are shown in Table 2. Both phase bin images had similar geometric shapes. However, the phase bin images with the higher target motion velocity (at phases 25% and 75%) had significantly larger target center position errors (3.0 to 3.8 mm). The position errors at phases 25% and 75% also indicated the time delay effect^25^ in a fast moving target image.^24^ The 20‐phase CT dataset showed similar target positions compared to the positions from 10‐phase dataset that are the most widely clinically used.

**Table 2:** Deviations in the target center position for regular motion in 10‐phase and 20‐phase CT datasets.

4D CT image type	Phase CT (%)	Phase image target center position (mm)	Expected target center position (mm)	Deviation (mm)
20‐phase	0	−14.7	−15	0.3
	25	3.3	0	3.3
	50	14.2	15	−0.8
	75	−3.8	0	−3.8
10‐phase	0	−14.7	−15	0.3
	25	3.0	0	3.0
	50	14.2	15	−0.8
	75	−3.4	0	−3.4

The target volume variations for regular and irregular motion patterns are shown in Tables 3 and 4, respectively. The variations for static CT and for phase bin CTs with regular motion were within 2.5%. The AIP and MIP CT datasets showed a volume reduction of −6.7% and −3.8%, respectively. For the irregular motions, ITV variations between AIP and MIP CTs (AIP/MIP) showed a smaller differences (−6.2 to −7.7%) for gated plans, and had a larger differences (−12.3 to −15.2%) for nongated plans.

**Table 3:** Variations in the target volume on CT dataset for the static and moving targets with regular breathing motion (static, 10‐phase, AIP and MIP CT datasets).

Target ball volume (V_T_, cm^3^)		4.19
CT type	GTV_static_ (cm^3^)	Variation (GTV_static_/V_T_)
Static CT	4.10	−2.1%

4D CT Phase	GTV_phase_ (cm^3^)	Variation (GTV_phase_/V_T_)
0%	4.20	0.3%
10%	3.90	−6.9%
20%	4.20	0.3%
30%	4.10	−2.1%
40%	4.10	−2.1%
50%	4.20	0.3%
60%	4.00	−4.5%
70%	4.00	−4.5%
80%	4.20	0.3%
90%	4.20	0.3%

Regular motion target volume (ITV_th_, cm^3^)		13.61
4D CT image type	ITV (cm^3^)	Variation (ITV/ITV_th_)
AIP	12.7	−6.7%
MIP	13.1	−3.8%

AIP, average intensity projection; GTV, gross tumor volumes; ITV, internal target volumes; MIP, maximum intensity projection.

**Table 4:** Variations in ITVs for AIP and MIP CT datasets in the irregular motion patterns for gated and nongated plans.

Phase range	Respiratory motion pattern	Image type	ITV (cm^3^)	ITV variation (AIP/MIP)
Gated 30%–70%	Slightly irregular	AIP	8.36	−7.73%
		MIP	9.06	
	Amplitude irregular	AIP	7.28	−7.61%
		MIP	7.88	
	Period irregular	AIP	10.12	−6.73%
		MIP	10.85	
	Period and amplitude irregular	AIP	7.33	−6.15%
		MIP	7.81	
Nongated 10%–90%	Slightly irregular	AIP	10.12	−12.31%
		MIP	11.54	
	Amplitude irregular	AIP	9.18	−12.99%
		MIP	10.55	
	Period irregular	AIP	10.2	−14.00%
		MIP	11.86	
	Period and amplitude irregular	AIP	8.19	−15.22%
		MIP	9.66	

AIP, average intensity projection; ITV, internal target volumes; MIP, maximum intensity projection.

### 3.2 Dosimetric variations

#### 3.2.1 Static target dose delivery

**Figure 4:**
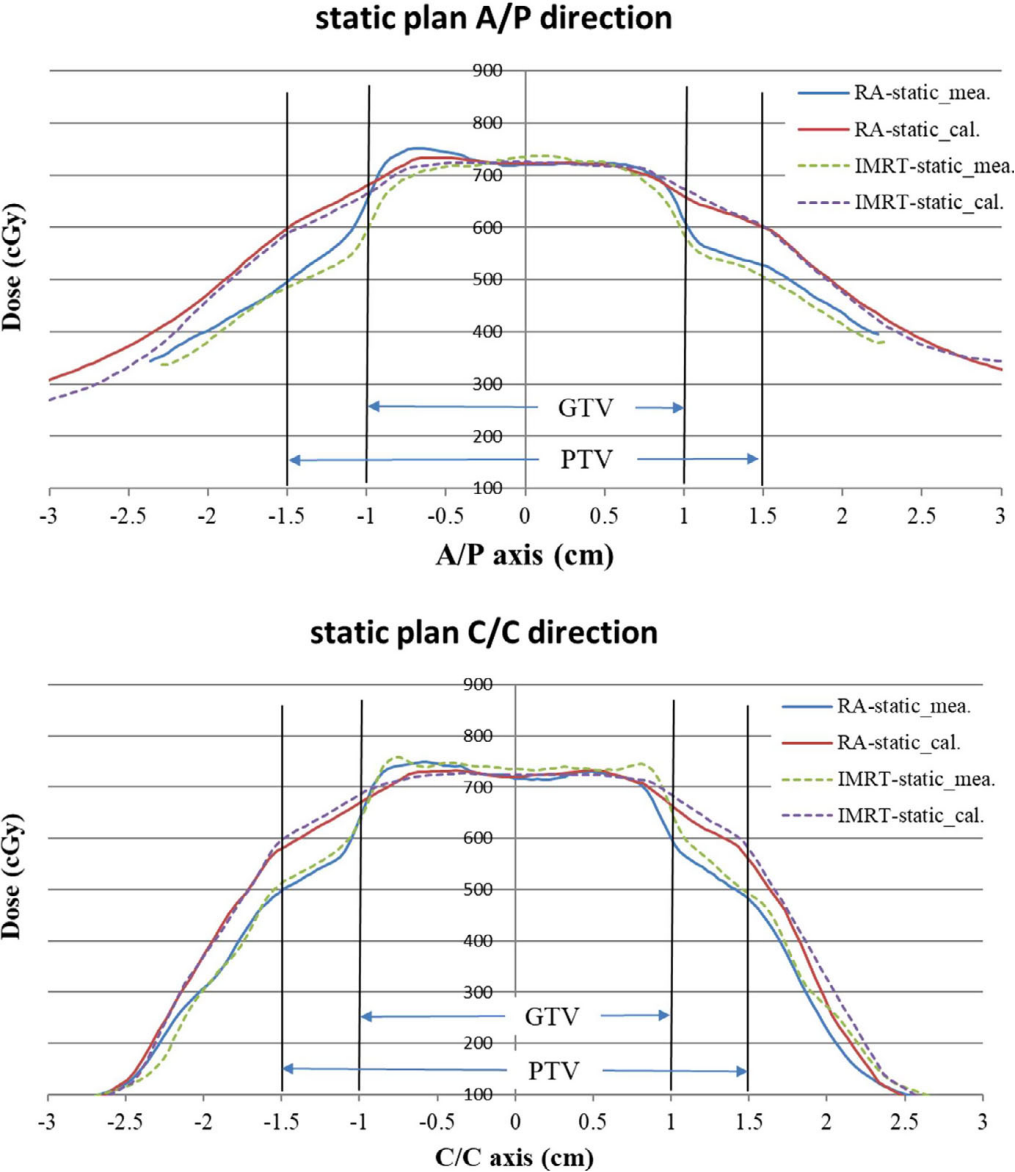
Dose profile comparison with the static CT between the treatment planning system calculations and film measurements in anterior/posterior (A/P) and cranial/caudal (C/C) directions.

Dosimetric comparisons showed a high passing rate with the static CT in the GTV for RA (99.5%) and IMRT (98.5%) plans. The dose profiles between the calculations and measurements in the anterior/posterior (A/P) and cranial/caudal (C/C) directions are shown in Fig. 4. The AAA algorithm over‐estimated the dose in the adjacent region of the target and the low density Styrofoam.

#### 3.2.2 Dynamic target dose delivery

The passing rates and statistical comparisons for different planning techniques, respiratory patterns, 4D CT resorting types, and dose delivery methods are listed in Table 5. AIP and MIP gated plans had average passing rates of 92.2% ± 5.3% and 85.8% ± 8.9%, respectively for different planning techniques and respiratory patterns, but no significant difference was observed. Nongated plans exhibited significantly lower, deviated passing rates. AIP and MIP plans were with passing rates of 43.6% ± 20.8% and 66.7% ± 36.4%, respectively (*P* < 0.05). For the gated plan, RA and IMRT had similar dosimetric results (*P* = 0.227). The dose accuracy to the dynamic target with the gated delivery technique was insensitive to breathing pattern irregularity.

**Table 5:** The passing rates (%) and statistical comparisons for different planning techniques (IMRT and RA), respiratory patterns (static and irregular), 4D CT datasets (AIP and MIP) and treatment delivery methods (gated and nongated plans).

Plan type	Static plan
RA	99.5
IMRT	98.5

Plan type	Gated plan		Nongated plan	
	A	B	C	D
	AIP	MIP	AIP	MIP
S‐I‐RA	87.3	80.7	63.0	86.7
S‐I‐IMRT	99.9	81.5	16.1	73.1
P‐I‐RA	91.8	92.4	57.9	18.5
P‐I‐IMRT	90.0	77.1	79.5	65.1
A‐I‐RA	96.6	86.6	22.1	31.1
A‐I‐IMRT	82.9	97.3	48.8	99.1
P+A‐I‐RA	91.1	72.6	33.3	72.1
P+A‐I‐IMRT	97.7	98.4	28.0	87.9
Avg.	92.2	85.8	43.6	66.7
STD	5.7	9.5	22.2	28.2
Statistic	A vs B	A vs C	B vs D	C vs D
*p*‐value	0.066	0.000	0.052	0.046

Abbreviations: AIP, average intensity projection; Amplitude irregular RA and IMRT plans (A‐I‐RA and A‐I‐IMRT); MIP, maximum intensity projection; Period and amplitude irregular RA and IMRT plans (P+A‐I‐RA and P+A‐I‐IMRT); Period irregular RA and IMRT plans (P‐I‐RA and P‐I‐IMRT); RA, rapid arc; Slight irregular RA and IMRT plans (S‐I‐RA and S‐I‐IMRT).

**Figure 5:**
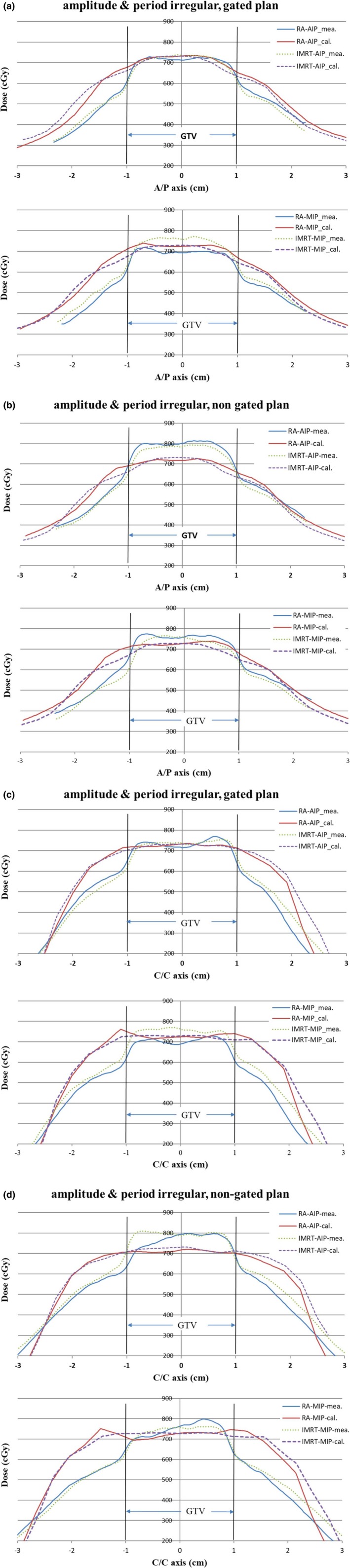
Dose profile comparison between calculations and measurements for the period‐ and‐amplitude irregular pattern with the target volume defined on average intensity projection and maximum intensity projection datasets, and delivered with the gated and nongated plans in A/P (a, b) and C/C axis (c, d).

The dose profile calculations and measurements for period‐ and amplitude‐irregular patterns with gated and nongated plans in A/P and C/C axis are shown in Fig. 5. As the results showed in the 2D gamma analysis, the dose profile comparisons in the target region were more consistent for the gated plans.

## 4 DISCUSSION

Irregular breathing causes variations in the patient motion pattern during imaging and treatment and it is a crucial problem when treating a mobile target, especially for hypo fractionation radiation therapy, which delivers a high fraction dose to a small lesion. The irregular breathing may result in an inappropriate estimation in the time‐correlated target position and the extent of the tumor motion (i.e., ITV). The interplay effect may be enhanced and then decrease the dose delivery accuracy. Therefore, this study quantitatively evaluated the irregularity effect on the geometry and dosimetry accuracy under four different irregular motion conditions.

How many respiratory‐correlated CT phases should be used to generate the geometric information for a mobile target in a 4D CT dataset? The number of CT phases is likely to affect the spatial accuracy for a moving target. However, increasing the number of datasets also increases the contouring workload. In this study, increasing the number of datasets to 20 phases in the regular motion model (i.e., sinusoidal waveform) did not show different target position information to that sorted in 10 phases (Table 2). The maximum position error was approximately 3.4 mm at phases of 25% and 75% with a motion velocity of about 23.56 mm/sec in the regular motion pattern. The positive and negative values of the position errors at phases 25% and 75% also indicated that there was a beam‐on imaging time delay of ~ 0.14 sec for the CT and RPM systems used in this study. This value is similar to Smith's report.^25^ The spatial accuracy of 4D CT images for a moving target seems affected mainly by the time delay effect but not the phase number. Planning CT for a moving target should avoid using only the phase bin image at the highest motion speed (e.g., phases of 25% and 75%).

The volumetric deviations for a moving target in the MIP or AIP images could lead up to 40% errors.^7^, ^8^, ^9^, ^23^, ^24^ In general, the deviation becomes more severe for a smaller and faster target motion. This study evaluated the volumetric deviations for static and different motion patterns. The volume deviations were small for static and phase CT images with most deviations less than 2.5% (Table 3). Larger volume deviations (less than 7%, underestimated) were observed in the MIP and AIP images for a regular motion pattern (Table 3). The target volume variations between MIP and AIP images were more significant in irregular motion patterns (Table 4), and the volume underestimation was more significant in the AIP image than the MIP. For a gated plan, the volume deviation was smaller compared to the nongated plan and depended on the gating interval. For clinical applications, adequate margins should be added to the ITV in the major motion directions based on the target size and the motion irregularity when using AIP or MIP images for ITV delineation. Based on this study, 1 mm margin is adequate to cover the volume deviation for target size <4 cm dia.

To avoid dosimetric analyses motion interference, IMRT and RA plans with static CT were measured and evaluated first. The dose in the GTV was consistent between the calculations and measurements for both plans (Fig. 4), with a passing rate higher than 98.5% (Table 5). However, as described in previous studies,^26^, ^27^, ^28^, ^29^ although the Varian AAA algorithm has shown a very small difference from the measured or Monte Carlo calculated doses within a homogeneous region, AAA is less accurate at a lung‐tissue interface and in particularly over‐estimates the dose to the lung region (Figs. 4 and 5). The target material (GTV) in this study was embedded in the very low density Styrofoam and this setup would enhance the dose inaccuracy in the region close to the target. The Varian Acuros XB (AXB) algorithm has been reported to have better agreement with Monte Carlo calculations than AAA at highly heterogeneous interfaces.^26^, ^27^, ^28^ However, AXB is under‐evaluated in our institution and was not included in this study. The main purpose of this study was to analyze the dose variations for a mobile target under different respiratory patterns. The dosimetric comparison between different calculation algorithms was beyond the scope of this study. Additionally, for the treatment of a moving target, the most important issue is to know if the target dose is adequate or not. In this study, the target doses in different motion conditions were measured directly and analyzed on the target (GTV) dose but not the peripheral dose.

The interplay effect and the variations in patient breathing patterns during the treatment may affect dose delivery accuracy for a moving target. The interplay effect results from the time‐related movement of internal structures and targets and dynamic dose delivery, leading to dosimetric variations between the planned and delivered dose distributions. The tighter the time‐related interactions, the more severe the interplay effect. As a consequence of this concept, interplay effect was larger for higher dose rate, low dose level, longer period time and longitudinal motion range, and fewer fractions. Respiratory gating with gated window between 30 and 70% at the end‐exhalation phase in this study had the moment with relatively small residual motion. The interplay effect impact might be reduced. In contrast to the gated plan, the nongated plan had larger ITV and included the faster motion phases that would increase the interplay effect. From Table 5, these enhancements in dosimetric variations were shown in nongated plans, although there were some nongated plans with good passing rates (e.g., A‐I‐IMRT and P+A‐I‐IMRT plans with MIP CT), but statistically analyzed the nongated plans, they still exhibited significantly lower and deviated passing rates.

The interplay effect could be averaged out after several fractions.^17^ However, this averaging could cause dose blurring at the field edges.^14^, ^15^ Our results demonstrated that taking a CBCT scan to reduce the positioning error, setting a proper margin to cover the full target motion range (ITV) and setup error, and using the gated dose delivery, the treatment in lung SBRT delivered by TrueBeam 6MVFFF beams did not compromise the GTV coverage (Table 5). This finding is consistent with other studies.^11^, ^12^, ^13^, ^14^, ^15^ In clinical patient treatment situations, as described in the Bo Zhao's report,^16^ tumor motion may change as the baseline shift that increases with the treatment time. They concluded that a high‐dose‐rate mode reduced the treatment time and thus reduced the interference in the baseline shifts. The concern in a higher dose rate with more interplay effect did not emerge in this phantom study. To mitigate the baseline shift in patient treatments, a high‐dose‐rate mode is an appropriate choice.

Four irregular motion models were created to evaluate the dose accuracy for a moving target under irregular respiratory patterns (Table 1). With gated delivery, the AIP plan passing rates under irregular respiratory patterns were slightly lower than that of static plans. The irregular motion patterns and plan techniques did not show a significant difference in the dose delivered to the target in the gated plans (Table 5). Treatment with phase‐based gated under irregular motion pattern may trigger the beam‐on and beam‐off signals at the wrong phase. The mis‐gating effect is managed using a margin. In this study, a 5 mm margin was added to the ITV to generate the PTV. This margin is more than 20% of the longitudinal ranges in the irregular motion models (Table 1). For gated plan, the ITV was further localized into the gated phases with relative smaller residual motion range. From the measurement results in this study, this margin is adequate to cover the mis‐gating effect.

For an intensity‐modulated plan, the beam intensity is modulated according to the dose constraints to the target and critical structures in the optimization process. In addition, the density distributions of these structures will affect the beam intensity map and the dose delivered. For a moving target in the AIP CT, as expected, the CT number of the ITV is “smeared” in these averaged images, and with a lower and wider density distribution along the direction of travel than that at the MIP and phase and static CT [Figs. 3(e) and 3(f)]. Different from the AIP image, the MIP image represents the highest intensity value in the volumetric dataset for the respective breathing phase. Glide‐Hurst et al.^30^ had shown that the AIP CT generated insignificant dose differences to a full 4D dose summation, and concluded that using the AIP CT can eliminate dose calculation on each 4D CT phase for lungs in clinical practice. In Tian et al.'s report,^31^ by comparing dosimetric characteristics between treatment plans calculated using free breathing, MIP and AIP CTs for lung SBRT patients. Their results have shown that FB and AIP plans were with similar mean effective depths but significantly different from that in MIP plans. They concluded that the AIP dataset is most favored for planning and dose calculation for lung SBRT. The dosimetric analysis in this study also showed that the AIP plans had a slightly higher passing rate than the MIP plans with the gated treatment technique (Table 5).

## 5 CONCLUSIONS

Faster target motion results in larger position errors in 4D CT image acquisition. Planning CT should avoid using only the phase bin image at the highest motion speed. The underestimation in ITV for a moving target was more significant in the AIP dataset than MIP, and is increasing with motion irregularity. This volumetric deviation is smaller for a gated plan than a nongated plan. The lung SBRT treatment delivered with the gated technique did not compromise GTV coverage and was insensitive to the breathing irregularities and plan techniques. Adequate margin should be made to cover the mis‐gating effect when using the phase‐based gating under irregular motion. Nongated plans had significantly lower and deviated passing rates than the gated plans.

## CONFLICT OF INTEREST

None of the authors has conflict of interest.
